# Analysis of Foveal Microvascular Abnormalities in Diabetic Retinopathy Using Optical Coherence Tomography Angiography with Projection Artifact Removal

**DOI:** 10.1155/2018/3926745

**Published:** 2018-09-18

**Authors:** Lun Liu, Jian Gao, Weili Bao, Chengyang Hu, Yajing Xu, Bingying Zhao, Jie Zheng, Lingling Fan, Yehuan Sun

**Affiliations:** ^1^Department of Epidemiology and Health Statistics, School of Public Health of Anhui Medical University, Hefei, Anhui, China; ^2^Department of Ophthalmology, The First Affiliated Hospital of Anhui Medical University, Hefei, Anhui, China

## Abstract

**Purpose:**

To analyze foveal microvascular abnormalities in different stages of diabetic retinopathy (DR) using optical coherence tomography angiography (OCTA) with projection artifact removal (PAR).

**Methods:**

We analyzed 93 eyes of 59 patients with diabetes—31 with no DR (no DR), 34 with mild to moderate nonproliferative DR (mild DR), and 28 with severe nonproliferative DR to proliferative DR (severe DR)—and 31 age-matched healthy controls. Sections measuring 3 × 3 mm^2^ centered on the fovea were obtained using OCTA. The area, perimeter, and acircularity index (AI) of the foveal avascular zone (FAZ), vessel density within a 300 *μ*m wide region of the FAZ (FD-300), and parafoveal vessel density in the superficial capillary plexus (SCP) and deep capillary plexus (DCP) were calculated using novel built-in software with PAR.

**Results:**

There was no statistically significant difference in the FAZ area (*p*=0.162). There was a statistically significant difference in the FAZ perimeter (*p*=0.010) and the AI (*p* < 0.001) between the four groups. There was a correlation between the AI and the increasing severity of DR (*p*=0.010). Statistically significant decreases of vessel density in the FD-300, SCP, and DCP were observed (all *p* < 0.001). There was a difference in parafoveal vessel density in the DCP between the healthy control eyes and the eyes with diabetes without DR (*p*=0.027). There was a significant correlation between vessel density and increasing severity of DR (*p* < 0.001).

**Conclusion:**

Compared with the FAZ area, AI allows a more helpful quantitative assessment of the changes in the FAZ. Vessel density determined using OCTA with PAR might be a useful parameter indicating the progression of DR. Parafoveal vessel density in the DCP after PAR might be a potential early biomarker of DR before appearance of clinically evident retinopathy and needs further investigation.

## 1. Introduction

Diabetic retinopathy (DR) is a major microvascular complication of diabetes and is a leading cause of acquired blindness in the population of working age worldwide [1, 2]. Chronic hyperglycemia leads to increased inflammation, hypoxia, and oxidative stress, all of which leads to changes in the microvessels of the retina [3]. Detection of these changes at the microvascular level during the different stages of DR is an area of increasing research interest that should provide important information regarding the perfusion status of the retina and the likelihood of developing more severe retinopathy. Dilated slit-lamp examination and fluorescein angiography of the fundus are the methods most commonly used to detect DR [4, 5]. However, these tests have limited ability to detect microvascular changes.

Optical coherence tomography angiography (OCTA) is a safe, rapid, and noninvasive technique for visualization of the retinal and choroidal microvessels with a resolution exceeding that of fundus fluorescein angiography (FFA) [6–9]. OCTA can show and quantify the superficial and deep vascular plexuses, impaired capillary perfusion, and neovascularization with high resolution [10–13]. OCTA also provides information on the dimensions and shape of the foveal avascular zone (FAZ), changes in which are most likely related to macular ischemia, especially in DR [14–17]. Enlargement of the FAZ area in DR has been reported [15, 18–20]; however, some studies did not detect differences in the FAZ area between eyes with diabetes without DR and control eyes without diabetes [21, 22]. There seems to be high variability in the FAZ area. A more helpful quantitative marker needs to be identified. Other studies have demonstrated the ability of OCTA to distinguish healthy eyes from eyes with varying levels of DR severity [13, 17, 23, 24], but these studies are limited by projection artifacts during visualization of the deeper retinal vascular structures. Projection artifacts can result in false measurements of vessel density and the FAZ borders in the deep capillary plexus (DCP) [24–26]. Accurate assessment of capillary nonperfusion in the retinal plexuses may be useful when monitoring diabetic retinopathy.

The aim of this study was to assess the characteristics of foveal microvascular abnormalities in different stages of DR using the recently developed AngioAnalytics software (version 2017.1.0.151) with projection artifacts removal (PAR) technology and new FAZ parameters. We studied several OCTA-based vascular parameters, including FAZ measurements and retinal vessel density. We compared the findings between patient groups, that is, controls, eyes with diabetes mellitus (DM) without DR, eyes with mild to moderate nonproliferative DR (NPDR), and eyes with the severe NPDR or proliferative DR (PDR).

## 2. Materials and Methods

This cross-sectional observational study was performed at the First Affiliated Hospital of Anhui Medical University, followed the tenets of the Helsinki and was approved by the Institutional Review Board of the First Affiliated Hospital of Anhui Medical University.

### 2.1. Participants

Ninety-three eyes of 59 patients with DM were examined. Eyes from patients with DM without DR and eyes with DR based on clinical assessment by retinal specialists were eligible for inclusion in the study. The exclusion criteria were glaucoma, uveitis, a high refractive error (more than 6 diopters), other retinal diseases, and cataract graded above nuclear opalescence. A healthy control group consisting of 31 eyes of 24 age-matched subjects without DM was also included. Each subject underwent comprehensive ocular examinations, including measurement of best-corrected visual acuity (BCVA), biomicroscopy examination of the fundus, color fundus photography, measurement of intraocular pressure, and OCTA. DR grades were based on the Early Treatment Diabetic Retinopathy Study classification endorsed in 2003 by the American Academy of Ophthalmology Guidelines Committee [27]. The eyes of the patients with diabetes were divided into three groups on the basis of their DR grades as determined by fundus photography and examinations as follows: a DM without DR (no DR) group, a mild to moderate NPDR (mild DR) group, and a severe NPDR to PDR (severe DR) group. BCVA was converted to the logMAR as described previously [28]. Eyes that had OCTA images with a scan quality index (SQI) less than 6 were excluded.

### 2.2. Angiographic Imaging

**Figure 1:**
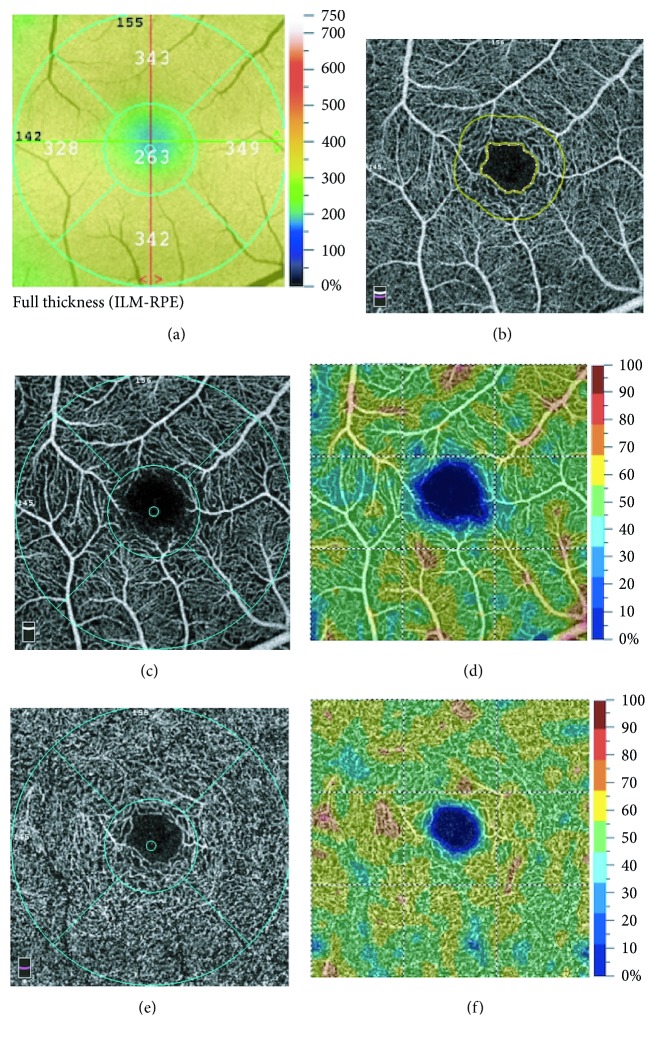
Measurement of optical coherence tomography angiography parameters on a 3 × 3 mm^2^. (a) The measurement of foveal retinal thickness. (b) The measurement of the foveal avascular zone. The following parameters were evaluated: area, perimeter, acircularity index, and vessel density of FD-300. (c, e) the vessel density in the superficial and deep capillary plexus. (d, f) Color maps show the vessel density in the superficial and deep capillary plexus.

An AngioVue OCTA device (Optovue, Inc., CA, USA) was used to obtain the angiography images [29]. This instrument performs 70,000 A-scans per second and acquires OCTA volumes consisting of 304 ∗ 304 A-scans over an area of 3 × 3 mm^2^. We obtained 3 × 3 mm^2^ sections centered on the fovea. The foveal thickness and SQI of each image were recorded. Foveal retinal thickness (FRT) was defined as the average retinal thickness within the central 1 mm diameter ring (Figure 1(a)).

We used the newly developed built-in AngioAnalytics software (version 2017.1.0.151) to obtain measurements for a series of parameters in the foveal area [29]. This software is an updated version that includes two important advances, that is, the PAR algorithm and improved FAZ parameters.

The FAZ was defined as the area encompassing the central fovea where there are no vessels. In the quantitative analysis of the FAZ, the area was evaluated as in previous studies but with the introduction of two additional parameters, that is, the perimeter and the acircularity index (AI) (Figure 1(b)). The device automatically outlines the boundary of the FAZ along the centermost capillaries, allowing the area and perimeter of this zone to be calculated. The AI is measured using the following equation: AI = perimeter calculated/standard circular perimeter of equal area. The AI becomes larger as the shape becomes less smooth or less round.

The proprietary three-dimensional PAR algorithm developed by Optovue removes projection artifacts from the entire OCTA volume on a per voxel basis, using information from the OCT and OCTA volume to differentiate the OCTA signal in situ from projection artifacts [29]. We obtained the vessel density of the full retina in a width of 300 *μ*m around the FAZ (FD-300) (Figure 1(b)) and the parafoveal vessel density for the superficial capillary plexus (SCP) (Figure 1(c)) and DCP (Figure 1(d)) using the built-in three-dimensional PAR software. The “parafovea” is defined as an annulus centered on the fovea with inner and outer ring diameters of 1 mm and 3 mm, respectively. Vessel density is a measurement of the proportion of pixels occupied by flowing vessels of the total pixels within the area of analysis (Figure 1(c), 1(d)). Error in automatic segmentation sometimes occurred; in these cases, we manually corrected the entire scan volume. The above parameters were used to assess impairment of ocular perfusion in the macular area.

### 2.3. Statistical Analysis

The data were assessed for normality using the Shapiro–Wilk test. All data are presented as the mean ± standard deviation (SD), median and interquartile range (IQR, 25th-75th percentile), or percentages as appropriate. Differences between the categorical data were assessed using the chi-square test or Fisher's exact test. Differences in the data obtained were assessed using Student's *t*-test, analysis of variance (ANOVA), or the Wilcoxon rank test, depending on their distribution. Since not all variances of the groups are equal, the nonparametric Kruskal–Wallis test with Bonferroni correction for multiple comparisons was used to compare values between groups. The Kendall tau correlation coefficient was used to examine correlations between the OCTA parameters and severity of DR. All the statistical analyses were performed using SPSS for Windows version 21.0 software (IBM Corp., Armonk, NY, USA). All statistical tests were considered significant when the *p* value was <0.05.

## 3. Results

### 3.1. Participant Demographics and Clinical Characteristics

One hundred twenty-four eyes of 83 subjects were included (31 eyes of 24 control subjects and 93 eyes of 59 patients with diabetes). The patient demographics, clinical characteristics, and SQI are reported in Table 1.

### 3.2. Analysis of the FAZ

**Figure 2:**
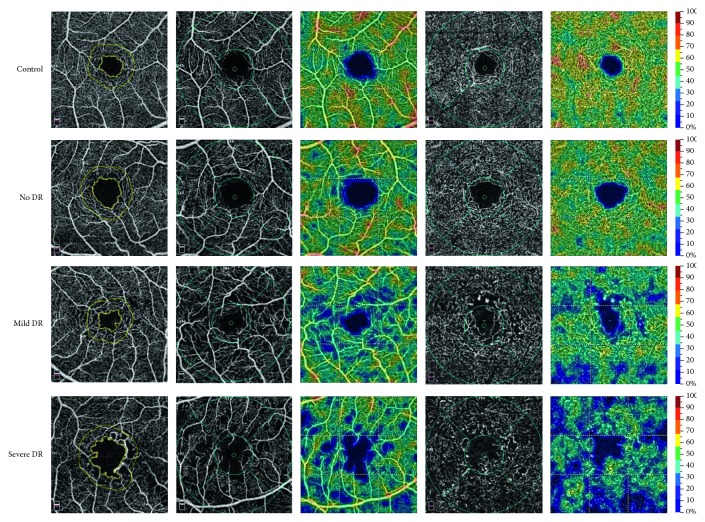
Representative samples of optical coherence tomography angiography parameters for each group (control, no DR, mild DR, and severe DR). The first column: area, perimeter, acircularity index of the foveal avascular zone increase from top to bottom, and vessel density in the FD-300 decrease. The second column: parafoveal vessel density in the superficial capillary plexus decreases from top to bottom. The fourth column: parafoveal vessel density in the deep capillary plexus decreases from top to bottom. The “parafovea” is defined as an annulus centered on the fovea with inner and outer ring diameters of 1 mm and 3 mm, respectively. The third column shows the vessel density in the superficial capillary plexus using color maps. The fifth column shows the vessel density in the deep capillary plexus using color maps. DR, diabetic retinopathy.

**Figure 3:**
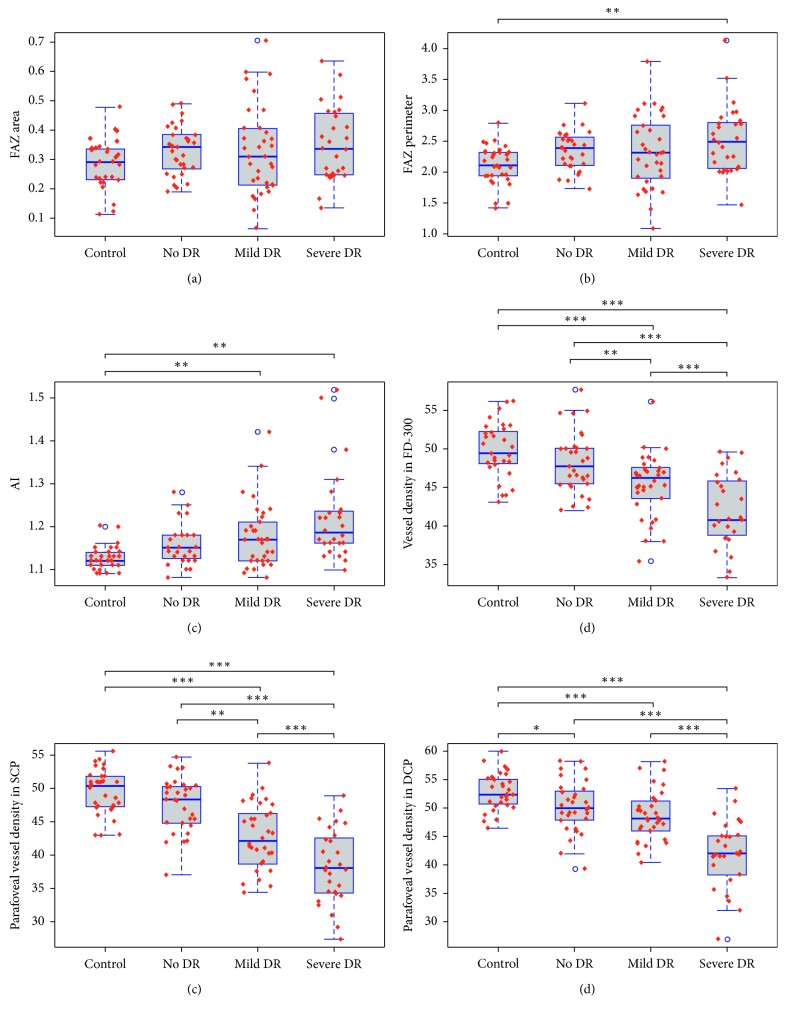
Boxplots of the foveal avascular zone area (a), perimeter (b), acircularity index (c), vessel density in the FD-300 (d), and the parafoveal vessel density in superficial (e) and deep (f) capillary plexus. ^*∗*^*p* < 0.05; ^*∗∗*^*p* < 0.01; ^*∗∗∗*^*p* < 0.001.

The first column in Figure 2 shows a representative sample of FAZ measurements with increasing DR severity. In the no DR group, the FAZ deviated from the gently undulating perimeter seen in the healthy controls. Breaks in the border, increased tortuosity, vessel loops, and budding of tortuous capillaries into the FAZ were also observed as the DR progressed. The average FAZ area, perimeter, and AI for the full retina in each study group (control, no DR, mild DR, and severe DR) are shown in Table 2. There was no statistically significant difference in the FAZ area between the four study groups (Table 2). Statistically significant differences in the FAZ perimeter and AI were observed between the four groups (*p* < 0.05). When post hoc multiple comparisons were performed, statistically significant increases in the FAZ perimeter (Table 2, Figure 3(b)) and AI (Table 2, Figure 3(c)) were observed in severe DR group when compared with the control group. When post hoc multiple comparisons were performed, statistically significant increases in the AI were observed in mild DR group when compared with the control group (Table 2, Figure 3(c)).

### 3.3. Analysis of Foveal Microcirculation Parameters

Figure 2 shows a representative sample of vessel density in the FD-300 (the first column) and parafoveal vessel density in the SCP (the second column) and DCP (the fourth column) with increasing severity of DR. Color maps were also used to show the changes in vessel density in the SCP (the third column) and DCP (the fifth column).  Table 3 shows the foveal microcirculation parameters in the FD-300, SCP, and DCP for each group. Statistically significant decreases in vessel density were observed in the FD-300, SCP, and DCP (Table 3). When post hoc multiple comparisons were performed, vessel density in the FD-300 and SCP was significantly higher in the control group than in the mild DR and severe DR groups (Figure 3(d) and 3(e)). Vessel density in the FD-300 and SCP was significantly higher in the no DR group than in the mild DR and severe DR groups (Figures 3(d) and 3(e)). Vessel density in the FD-300 and SCP was significantly higher in the mild DR group than in the severe DR group (Figures 3(d) and 3(e)). The vessel density in the DCP was significantly higher in the control group than in the other three groups (Figure 3(f)). Vessel density in the DCP was significantly higher in the no DR and mild DR groups than in the severe DR group (Figure 3(f)). Vessel density in the DCP was significantly higher in the mild DR group than in the severe DR group (Figure 3(f)).

### 3.4. Correlations between OCTA Parameters and Severity of DR

In Kendall tau correlation coefficient analysis, we observed significant correlations between severity of DR and vessel density in the FD-300, SCP, and DCP (*p* < 0.05) (Table 4). Retinal vessel density measured in the FD-300, SCP, and DCP decreased significantly with increasing severity of DR. The AI also showed a significant trend of increase with increasing severity of DR (*p* < 0.05) (Table 4).

## 4. Discussion

In this study, we quantified and analyzed the foveal microvascular abnormalities in different stages of DR using OCTA. Previous studies have reported the changes in the capillaries of the macular area using conventional fundus photography and FFA and suggested that the foveal microvascular abnormalities might be useful when evaluating the progression of DR [4, 5, 30]. However, conventional fundus photography had limited image resolution and FFA has potentially serious side effects because of injection of fluorescent dye. New technologies have been evaluated as an alternative to FFA, and OCTA, which is rapid to perform and noninvasive, has shown the greatest potential in this field [14–17,31]. Good reproducibility and repeatability of FAZ measurements in both healthy eyes and eyes with retinal diseases have been reported recently [32]. Furthermore, no statistically significant difference in FAZ perimeter, AI, or vessel density was found between measurements [33]. These results, together with the easy operation of the device, short acquisition time, and avoidance of potentially phototoxic blue light, suggest that OCTA holds promise as a tool for monitoring ocular pathology and detecting early disease [34].

In this study, we used AngioAnalytics software that improves and streamlines the interpretation of OCTA data by removing projection artifacts from the entire OCTA volume and introduces new FAZ parameters for analysis.

Previous studies have shown enlargement of the FAZ area in eyes with DR [15,25–27]. However, another study did not show a difference in the FAZ area between patients with DM without DR and nondiabetic controls [21]. FFA did not identify significant enlargement of the FAZ but found significant enlargement of the parafoveal intercapillary area in the SCP [22]. Our results showed no significant change in the FAZ area during progression of DR. This could be related to the high variability of the FAZ area. There is considerable variation in the FAZ area between normal individuals and patients with diabetes [35–37]. There was a significant overlap between the healthy and diseased groups, which limited the sensitivity and specificity of the FAZ area for identifying and staging disease [35–37]. The quantitative difference in the FAZ area did not correspond to morphologic changes in the regularity and appearance of the FAZ in the concomitant qualitative analysis [38].

Numerous qualitative studies have demonstrated that FAZ becomes more acircular in DR, with greater effects on the perimeter than on the area [19,39,40]. However, few studies have attempted to quantify the irregularity of the FAZ and correlate this metric to the stages of DR. In our study, we found that the FAZ in DR deviated from the gently undulating perimeter found in healthy controls and became apparent in eyes with serious disease. Breaks in the border, increased tortuosity, vessel loops, and budding of tortuous capillaries into the FAZ were also observed and were similar to the findings in previous qualitative studies [39]. On further quantitative evaluation, we found statistically significant trends with increasing severity of DR. AI may be more useful for monitoring of DR than the FAZ area.

Our next finding was that vessel density showed a strong correlation with the severity of DR, which is consistent with previous studies [23]. The FD-300 was the area that corresponded to the juxtafoveal region where capillary loss has been described in previous studies in patients without DR [18, 20], but was not observed in our study population. Nesper et al. [23] reported finding no difference in vessel density (SCP or DCP) between healthy control eyes and the eyes of patients with DM without DR. Interestingly, we observed a significant difference in vessel density only at the DCP but not at the SCP when comparing the eyes of healthy controls with those of patients with DM without DR. This may be explained by projection artifacts. Projection artifacts, which come from fluctuating shadows cast by blood cells flowing in the more superficial vessels, affect visualization of the deeper retinal vascular structures [41–43]. These projection artifacts can be picked up by most OCTA technologies as false flow and cause superficial vascular networks to be duplicated on deeper slabs on en face angiograms. Projection artifact can give false impressions of the density of vessels, especially in evaluation of the DCP [24–26,41]. With PAR, projection artifacts can be removed from the normally avascular outer retinal slab while preserving the density and continuity of the intermediate and deep retinal capillary plexuses [25, 29]. In this study, as a result of advances in OCTA software, we were able to perform a more refined analysis of the DCP because of the removal of projection artifacts. The FD-300 was the vessel density of the full retina, not only the SCP or DCP in a 300 *μ*m around the FAZ.

It is important to note that in this study, vessel density in the DCP decreased significantly in the eyes of patients with DM without DR. This metric measured by OCTA with PAR may help identify vascular changes before the onset of funduscopically visible disease. This is a finding that deserves further study as a potential early biomarker of DR before clinically evident retinopathy.

The potential limitations of this study are its cross-sectional design and evaluation of FAZ area, which may vary consistently even in normal individuals. A prospective and longitudinal study is needed in the future. Another limitation may be the automated preset algorithm in the AngioVue system that only segments two retinal capillary plexuses, that is, the SCP and DCP. Some studies have suggested that the SCP, middle capillary plexus (MCP), and DCP show different areas of nonperfusion in eyes with DR [44, 45]. Therefore, the SCP, MCP, and DCP should be assessed quantitatively in future studies. The strength of our study was the PAR in evaluation of the DCP. Segmentation errors and decreased SQI may impact the automatically generated vessel density and FAZ metrics. To avoid this problem, we manually corrected the entire scan volume and excluded eyes that had OCTA images with an SQI less than 6. No significant differences in SQI were found between the four groups.

## 5. Conclusions

This study introduces AI as a marker of changes in the FAZ and shows that it may be a more helpful imaging biomarker than FAZ area for monitoring the progression of DR. We found a significant correlation between vessel density and disease severity in eyes with DR. Furthermore, the parafoveal vessel density in the DCP determined by OCT with PAR may serve as an early indicator for subclinical stages of DR and needs further investigation.

## Figures and Tables

**Table 1:** Demographic and clinical characteristics of the study participants.

	Control	No DR	Mild DR	Severe DR	*p* value
Patients, *n*	24	17	21	21	
Eyes, *n*	31	31	34	28	
Sex					0.151
** **Female, *n* (%)	19 (79.2)	14 (82.4)	12 (57.1)	12 (57.1)	
** **Male, *n* (%)	5 (20.8)	3 (17.6)	9 (42.9)	9 (42.9)	
Age, *y*, mean ± SD	53.2 ± 8.53	56.3 ± 8.21	53.2 ± 9.94	50.4 ± 7.51	0.226
Disease duration, *y*, mean ± SD	—	6 (1–7)	10 (7–20)	10 (8.5–15)	0.001
BCVA, logMAR, mean ± SD	0 (0–0.1)	0 (0–0.1)	0.1 (0.0–0.2)	0.4 (0.2–0.7)	<0.001
FRT, *µ*m, mean ± SD	248.0 ± 17.53	248.0 ± 14.54	246.3 ± 30.11	309.1 ± 120.44	0.001
SQI, mean ± SD	7.26 ± 0.68	7.00 ± 0.68	7.06 ± 0.69	6.82 ± 0.82	0.141

**Table 2:** Comparisons of measurements at the foveal avascular zone in the four study groups.

	Control *n*=31	No DR *n*=31	Mild DR *n*=34	Severe DR *n*=28	F/H value	*p* value
FAZ area, mm^2^, mean ± SD	0.28 ± 0.08	0.33 ± 0.08	0.33 ± 0.15	0.35 ± 0.13	5.135	0.162^a^
FAZ, perimeter, mm, mean ± SD	2.10 ± 0.32	2.34 ± 0.32	2.33 ± 0.58	2.52 ± 0.54	11.277	0.010^b^
AI, mean ± SD	1.13 ± 0.03	1.16 ± 0.05	1.18 ± 0.07	1.22 ± 0.10	26.794	<0.001^c^

^a^Multiple comparisons are tested using the Kruskal–Wallis test; ^b^multiple comparisons are tested using the Kruskal–Wallis test, severe DR > control (*p*=0.007, after Bonferroni correction); ^c^multiple comparisons are tested using the Kruskal–Wallis test, mild DR > control (*p*=0.008, after Bonferroni correction) and severe DR > control (*p* < 0.001, after Bonferroni correction).

**Table 3:** Comparisons of the foveal microcirculation parameters between the four study groups.

	Control *n*=31	No DR *n*=31	Mild DR *n*=34	Severe DR *n*=28	F/H value	*p* value
Vessel density						
FD-300, %, mean ± SD	49.82 ± 3.55	48.21 ± 3.93	45.33 ± 4.12	41.87 ± 4.67	21.683	<0.001^d^
Parafoveal vessel density						
SCP, %, mean ± SD	49.47 ± 3.41	47.56 ± 4.08	42.57 ± 4.94	38.26 ± 5.52	58.526	<0.001^e^
DCP, %, mean ± SD	52.85 ± 3.22	50.26 ± 4.54	48.58 ± 4.34	41.86 ± 5.93	30.643	<0.001^f^

^d^Multiple comparisons are tested using one-way ANOVA with a post hoc test, control > mild DR (*p* < 0.001), control > severe DR (*p* < 0.001), no DR > mild DR (*p*=0.005), no DR > severe DR (*p* < 0.001), and mild DR > severe DR (*p* < 0.001); ^e^multiple comparisons are tested using the Kruskal–Wallis test, no DR > severe DR (*p* < 0.001, after Bonferroni correction), control > severe DR (*p* < 0.001, after Bonferroni correction), no DR > mild DR (*p*=0.003, after Bonferroni correction), mild DR > severe DR (*p* < 0.001, after Bonferroni correction), and control > mild DR (*p* < 0.001, after Bonferroni correction); ^f^multiple comparisons are tested using one-way ANOVA with a post hoc test, control > no DR (*p*=0.027), control > mild DR (*p* < 0.001), control > severe DR (*p* < 0.001), no DR > severe DR (*p* < 0.001), and mild DR > severe DR (*p* < 0.001).

**Table 4:** Correlations between OCTA parameters and severity of DR.

	No DR *n*=31	Mild DR *n*=34	Severe DR *n*=28	Correlation coefficient	*p* value
FAZ area, mm^2^, mean ± SD	0.33 ± 0.08	0.33 ± 0.15	0.35 ± 0.13	0.017	0.832
FAZ, perimeter, mm, mean ± SD	2.34 ± 0.32	2.33 ± 0.58	2.52 ± 0.54	0.091	0.260
AI, mean ± SD	1.16 ± 0.05	1.18 ± 0.07	1.22 ± 0.10	0.213	0.010
Vessel density					
FD-300, %, mean ± SD	48.21 ± 3.93	45.33 ± 4.12	41.87 ± 4.67	−0.384	<0.001
Parafoveal vessel density					
SCP, %, mean ± SD	47.56 ± 4.08	42.57 ± 4.94	38.26 ± 5.52	−0.490	<0.001
DCP, %, mean ± SD	50.26 ± 4.54	48.58 ± 4.34	41.86 ± 5.93	−0.441	<0.001

## Data Availability

Researchers can access the data supporting the conclusions of this study through the authors. Lun Liu (liulundoc@126.com) and Jian Gao (shuijinglovegj@126.com) can be contacted to request the data.
